# Genomic alterations in KMT2 family predict outcome of immune checkpoint therapy in multiple cancers

**DOI:** 10.1186/s13045-021-01050-0

**Published:** 2021-03-02

**Authors:** Peng Zhang, Yixuan Huang

**Affiliations:** Division of Immunotherapy, Institute of Human Virology, University of Maryland School of Medicine, 725 W. Lombard St., Baltimore, MD 21201 USA

**Keywords:** Immune checkpoint therapy, Biomarker, KMT2 gene family, Lung cancer, Melanoma

## Abstract

Immune checkpoint therapy (ICT) can produce durable antitumor responses in various cancer types; however, the responses are not universal, and the predictive biomarkers are urgently needed. Growing evidence points to a link between epigenetic regulation and anti-tumor immunity, while clinical data on the association of genomic alterations in transcriptional dysregulation-related genes and ICT clinical benefit are lacking. Histone–lysine *N*-methyltransferase 2 (KMT2) family proteins methylate lysine 4 on the histone H3 tail at important regulatory regions in the genome and thereby impart crucial functions through modulating chromatin structures and DNA accessibility, which is associated with tumorigenesis, mutagenesis, and immune tolerance in various cancers, indicating its possible correlation with the output of immune checkpoint therapy. We hypothesized that genomic mutations in the KMT2 family have the potential to be a novel predictor of immunotherapeutic efficacy. Through integrative cancer genomic analyses of baseline tumor tissues from multiple cohorts involving immunotherapeutic patients, we uncovered a remarkable correlation between KMT2 family mutation and better immune checkpoint therapy responses in multiple patient cohorts. Then, we gathered all the independent ICT-treated datasets as a combination cohort consisted of 418 patients. Significant enrichment of KMT2 family genomic alterations in responding tumors was observed (odds ratio = 2.60, *P* value = 1.67e−04). This work distinguished the mutations in the KMT2 family as a potential predictor of favorable ICT response in multiple cancers, highlighting the importance of genomic profiling in immunotherapy.

## To the Editor,

**Fig. 1:**
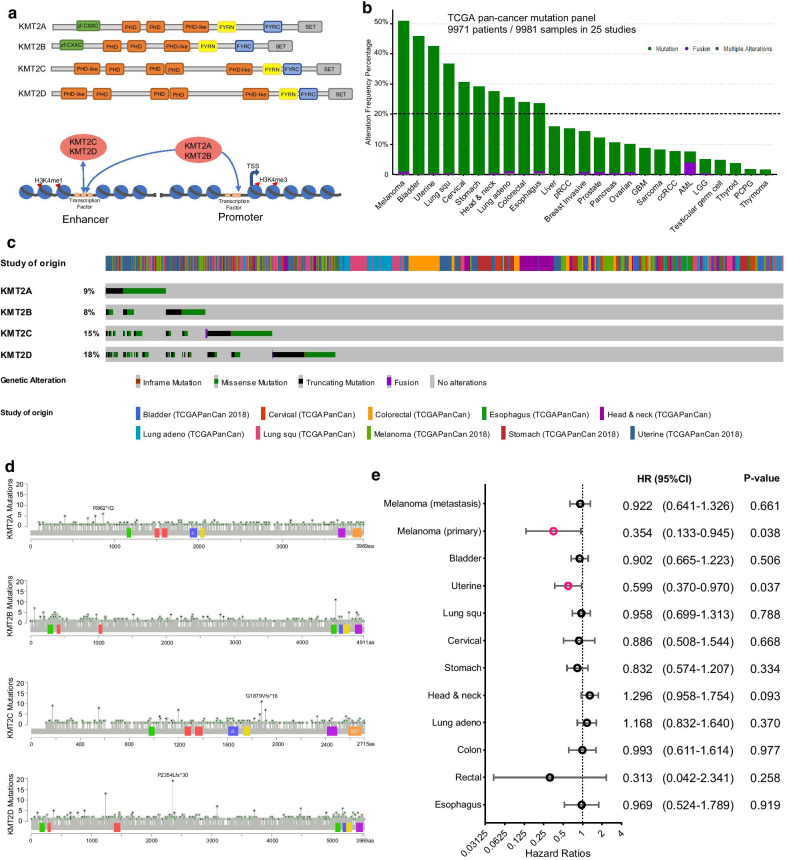
The pan-cancer landscape of KMT2 family mutations across human tumors. **a** Schematic representation of the domain structures (top) and transcription regulatory regions (bottom) for KMT2 family genes are shown. **b** The proportion of KMT2 family mutated tumors identified for each cancer type with alteration frequency in TCGA pan-cancer cohorts. **c** Mutation patterns (as Oncoprint schematics) across the KMT2 family hyper-altered patient cohorts from the TCGA database. Truncating mutations included nonsense, nonstop, splice site mutations, and frameshift insertion and deletion; Non-truncating mutations included missense mutations and inframe insertion and deletion. **d** Lollipop plot showing the loci distribution of mutations across the KMT2 family hyper-altered patient cohorts from the TCGA database. **e** Hazard ratio (HR) estimates for overall survival in TCGA pan-cancer cohorts. HRs compare KMT2 family mutation and wildtype status by using Cox regression. Plotting symbols give point estimates of HR and horizontal bars give 95% CIs

Members of the KMT2 family methylate histone H3 on lysine 4 (H3K4) to promote genome accessibility and transcription, which is required for early epigenetic decisions during development and contributes to the methylation of bivalent promoters [1, 2] (Fig. 1a). In recent years, a multitude of tumor exome sequencing studies has revealed that KMT2 family are mutated in a significant percentage of a large variety of malignancies and strongly linked to tumorigenesis, mutagenesis, and immune tolerance [3, 4]. The potential association of epigenetic dysregulation caused by KMT2 family mutations and cancer immunotherapy benefit has motivated us to investigate the precise function of these genomic alterations in predicting immune checkpoint therapy outcomes in human cancers. The KMT2 gene family contained three subgroups (within two paralogues in each subgroup): *KMT2A* and *KMT2B*; *KMT2C* and *KMT2D*; and *KMT2F* and *KMT2G*, which is highly conserved throughout eukaryotes [3] (Fig. 1a). In tumor cells, four members of the KMT2 family (*KMT2A*, *KMT2B*, *KMT2C*, and *KMT2D*) are among the most frequent genomic alterations in different cancer types [3, 4]. Focusing on these genes, we investigated the somatic mutation frequency of the pan-cancer cohorts that consisted of 9,981 patients across 25 cancer types based on The Cancer Genome Atlas (TCGA) database. Although the genomic alteration level of the KMT2 family in human cancers was overall high, several cancer types (such as melanoma, blander, uterine, and lung carcinomas) showed a dramatically higher number of somatic alterations when compared with other cancers. As shown in Fig. 1b, given the background DNA aberration frequencies in a specific cancer type, the KMT2 family genes were identified as hyper-altered in 10 cancer types (mutation frequency ranging from 23 to 51%). Regarding the mutational profile for individual genes of the KMT2 family (Fig. 1c), *KMT2D* (18%) ranked the highest mutation frequency, followed by *KMT2C* (15%), *KMT2A* (9%), and *KMT2B* (8%) in these KMT2 hyper-altered patient cohorts. Nevertheless, there was no hotspot mutation specifically enriched in any individual members of the KMT2 gene family was observed (Fig. 1d). Then, we systematically collected and analyzed the clinical data of patient cohorts to evaluate the prognosis function of KMT2 family mutations at the pan-cancer level. As shown in Fig. 1e, we found that mutations in KMT2 gene family were positively correlated with better overall survival (OS) only in melanoma (primary) and uterine corpus endometrial carcinoma, while no association was observe in most of hyper-altered cancer types. Besides, to gain insights into the molecular mechanisms underlying the mutations of KMT2 gene family in cancer cells, we analyzed differential gene expression patterns between the KMT2 mutant and wild-type samples in TCGA metastasis melanoma patient cohort (Additional file 3: Fig. S1). The function terms of nuclear division and cell cycle are enriched in the patient subgroup with KMT2 mutation, while the activity of vessel development and morphogenesis functions is down-regulated. These results demonstrated that KMT2 mutations did not have a direct effect on tumor immunology but could influence the reorganization of the cancer microenvironment (for example, angiogenesis).

**Fig. 2:**
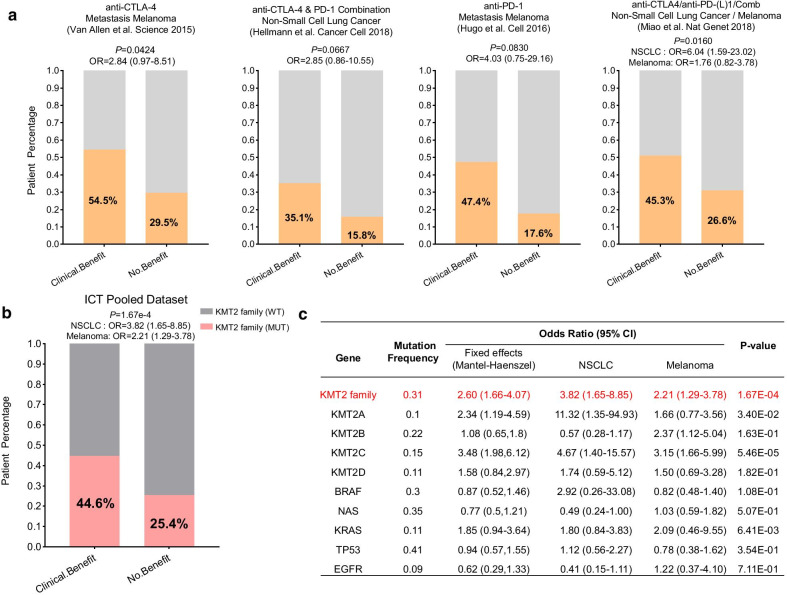
KMT2 family mutations in the baseline tumor tissue correlate with favorable responses to ICT. **a** Histogram depicting proportions of harboring KMT2 family mutations in clinical benefit and non-benefit groups of different ICT-treated patient cohorts. **b** Pooled estimates of odd ratios of KMT2 family mutation as a predictive marker for ICT, and **c** compared with other known predictive gene mutations. The individual odds ratio of the pooled dataset was calculated based on the Mantel–Haenszel model, and the *P* value was determined by Fisher's exact test

Then, to investigate whether the genomic alterations in the KMT2 gene family were related to the response to ICT, the clinical cohorts with response annotation and matched mutational data from published studies were collected and consolidated [5–8]. To verify our hypothesis, we divided these public accessible immunotherapy-treated patient cohorts into the KMT2-WT and KMT2-MUT subgroups and analyzed the correlation between the KMT2 family mutation status and the clinical benefit of ICT. As shown in Fig. 2a, all patient cohorts showed a trend that the patients harboring KMT2 family mutation had a better durable clinical benefit (DCB) and most of them were statistically significant. Potential clinical implications are stratified by the level of evidence that a specific molecular alteration has biologic and oncogenic effects. So we further checked whether predicted pathogenic variants were associated with clinical outcomes. Based on the prediction results by OncoKB [11], the mutations of the KMT2 gene family could be divided into two categories: “Putative Driver” and “Putative Passenger.” As shown in Additional file 3: Fig. S2, we did not observe a significant correlation between the predicted driver variants and ICT outcome (partly due to the very limited number of somatic driver mutations in the KMT2 family). But that indicated the pathogenic driver variants may not necessarily associate with response to ICT. Next, to characterize the tumor immune microenvironment of KMT2 mutated tumors, we compared the tumor immunogenicity and checkpoint gene expression (PD-L1) between KMT2 mutant and wild-type samples. Tumor mutation burden (TMB) level was significantly higher in KMT2-MUT tumors compared with that in the KMT2-WT tumors in all ICT cohorts (Additional file 3: Fig. S3A), indicating that mutations of the KMT2 gene family were positively correlated with boosted tumor immunogenicity. Interesting, there is no difference in PD-L1 mRNA expression observed between KMT2 mutant and wild-type tumors (Additional file 3: Fig. S3B), which revealed KMT2 mutations were not strongly associated with enhanced immune checkpoint expression level. Besides, the mutational patterns (Additional file 3: Fig. S4) of the KMT2 gene family in ICT patient cohorts were very similar to the mutation profile of patients from the TCGA cohorts.

To further validate the predictive function of KMT2 mutation on ICT clinical response, a combined cohort of 418 ICB-treated patients that were pooled from all the publicly available studies was gathered (Fig. 2b), which consisted of two cancer types: melanoma (*N* = 287), and non-small cell lung cancer (NSCLC) (*N* = 131). In this pooled cohort, the clinical benefit of immune checkpoint therapy was more prominent in the KMT2-MUT group than that in the KMT2-WT group (44.6% vs 25.4%, M-H OR 2.60, *P* value = 1.67e−04). Even compared with other known oncogenes, most of which had been reported to affect the efficacy of immunotherapies [9, 10], KMT2-MUT remained the most significant predictor for ICT clinical benefit. The odds ratios for all the high-frequency mutated genes (> 20% in both NSCLC and Melanoma) are summarized in Additional file 2: Table S1.

Collectively, we conducted an integrative study to evaluate the prevalence of KMT2 family mutation and its correlation with preliminary response to ICT in multiple cancer types. Our results indicated that genomics alteration in the KMT2 family was more likely to be a positive predictive biomarker for the clinical benefit of cancer immunotherapy, which might aid the identification of ideal candidates and tailor optimal immunotherapeutic strategies. Nevertheless, as this is a retrospective study and the sample size is relatively small, these conclusions need to be interpreted with caution. We still lack sufficient predictive biomarkers to guide patient selection in clinical use. Further investigations in a larger cohort of patients receiving immune checkpoint therapies are needed to determine its potential use as a clinical biomarker for cancer immunotherapy responsiveness.

## Supplementary Information

**Additional file 1**. Supplementary materials and methods.

**Additional file 2: Table S1**. The estimated odds ratio of high-frequency gene mutations as predictive marker in ICT pooled cohort

**Additional file 3: Figure S1**. KMT2 mutations correlated with tumor microenvironment reorganization in metastasis melanoma. (A) Volcano plot of mRNA expression changes between metastasis samples harboring KMT2 mutation and wild-type. The x-axis specifies the fold-changes (FC), and the y-axis specifies the negative logarithm to the base 10 of the adjusted P values. Gray vertical and horizontal dashed lines reflect the filtering criteria. Red and blue dots represent genes expressed at significantly higher or lower gene levels, respectively. (B) Functional enrichment analysis based on gene ontology terms for differential expressed genes. **Figure S2**. No significant correlation was observed between predicted KMT2 driver mutations and ICT clinical outcomes. Histogram depicting proportions of harboring KMT2 family predicted driver mutations in clinical benefit and non-benefit groups of different ICT-treated patient cohorts. The P value and odds ratios were determined by Fisher's exact test. **Figure S3**. Association analysis between KMT2 family mutations and tumor immunogenicity in ICT cohorts. (A) The distribution plot of the tumor somatic mutation burden level between KMT2-MUT and KMT2-WT samples in all the ICT patient cohorts. (B) The distribution plot of the PD-L1 expression level between KMT2-MUT and KMT2-WT samples in all the ICT patient cohorts. The P value was determined by the Mann–Whitney test. **Figure S4**. The mutation pattern of KMT2 genes in ICT patient cohorts. Oncoprint schematics revealed the mutational pattern of the KMT2 family mutations in all the ICT patients cohorts involved in this study. Truncating mutations included nonsense, nonstop, splice site mutations, and frameshift insertion and deletion; Non-truncating mutations included missense mutations and inframe insertion and deletion.

## Data Availability

The materials of patient cohorts used for the current study were publicly available and can be assessed by the TCGA database (https://portal.gdc.cancer.gov/, https://www.cbioportal.org/). The processed data and analysis codes are available upon reasonable request from the corresponding author.

## Abbreviations

ICT: Immune checkpoint therapy
TCGA: The Cancer Genome Atlas
OS: Overall survival
DCB: Durable clinical benefit
TMB: Tumor mutation burden
NSCLC: Non-small cell lung cancer
