# Supplementary material for: Genomic alterations in KMT2 family predict outcome of immune checkpoint therapy in multiple cancers
Source: J Hematol Oncol. 2021 Mar 2;14:39. doi: 10.1186/s13045-021-01050-0 (PMC7927217; doi:10.1186/s13045-021-01050-0)
Supplement: Supplementary file 1 — Additional file 1. Supplementary materials and methods. [file 13045_2021_1050_MOESM1_ESM.pdf]

Supplementary Methods

TCGA pan-cancer patient cohort

The genomic alteration data (somatic mutations) and clinical annotation (including overall survival time) of 9981 samples across 25 cancer types were obtained from public repositories based upon data generated by the TCGA database (http://cancergenome.nih.gov/, February 2020). All the somatic mutations of the KMT2 gene family used in this work are somatic coding mutations (including protein-altering and splice). The majority of these mutations are missense and truncating mutations, and there are also a few fusion and inframe mutations involved. The predicted biological effect of mutations was determined by the OncoKB database [1].

Immunotherapy patient cohort

The genomic sequencing data and clinical annotation (DCB) of selected ICT-treated patient cohorts[2–5], which contained a total of 457 tumor samples across 5 cancer types, were accessed from the cBioPortal for Cancer Genomics database (http://www.cbioportal.org/index.do, February 2020).

Quantification and statistical analysis

The specific tests used to analyze each set of experiments are indicated in the figure legends. For the analysis of the contingency tables, Fisher's exact test was used to calculate the significance. To perform a patient's cohort-based analysis on the overall survival rate (5-year), the prognostic value of discrete variables was estimated by using the Kaplan-Meier survival curves, and the log-rank test was employed to estimate the significance among different survival curves. All statistical calculations were performed using GraphPad Prism software (GraphPad Software, San Diego, California) or R software (https://www.r-project.org/).

Differential Expression and Functional Enrichment Analysis

The genome-wide differential expression analysis was performed by using the DESeq2 package[6]. A gene was considered differentially expressed when the absolute difference of FPKM (Fragments per kilo base per million mapped reads) value between compartments was greater than 1, the log2 fold-change was greater than 1 and the adjusted P-value (FDR) was less than 0.05. The functional enrichment analysis based on gene ontology annotation terms was run on differentially expressed genes that were identified in this work, using the WEB-based Gene Set Analysis Toolkit[7].

References

1. Chakravarty D, Gao J, Phillips SM, Kundra R, Zhang H, Wang J, et al. OncoKB: A Precision Oncology Knowledge Base. JCO Precis Oncol. 2017;2017.

2. Miao D, Margolis CA, Vokes NI, Liu D, Taylor-Weiner A, Wankowicz SM, et al. Genomic correlates of response to immune checkpoint blockade in microsatellite-stable solid tumors. Nat Genet. 2018;50:1271–81.

3. Van Allen EM, Miao D, Schilling B, Shukla SA, Blank C, Zimmer L, et al. Genomic correlates of response to CTLA-4 blockade in metastatic melanoma. Science. United States; 2015;350:207–11.

4. Hugo W, Zaretsky JM, Sun L, Song C, Moreno BH, Hu-Lieskovan S, et al. Genomic and Transcriptomic Features of Response to Anti-PD-1 Therapy in Metastatic Melanoma. Cell. 2016;165:35–44.

5. Hellmann MD, Nathanson T, Rizvi H, Creelan BC, Sanchez-Vega F, Ahuja A, et al. Genomic Features of Response to Combination Immunotherapy in Patients with Advanced Non-Small-Cell Lung Cancer. Cancer Cell. 2018;33:843-852.e4.

6. Love MI, Huber W, Anders S. Moderated estimation of fold change and dispersion for RNA-seq data with DESeq2. Genome Biol. England; 2014;15:550.
7. Liao Y, Wang J, Jaehnig EJ, Shi Z, Zhang B. WebGestalt 2019: gene set analysis toolkit with revamped UIs and APIs. Nucleic Acids Res. 2019;47:W199–205.
