# Supplementary material for: Genomic alterations in KMT2 family predict outcome of immune checkpoint therapy in multiple cancers
Source: J Hematol Oncol. 2021 Mar 2;14:39. doi: 10.1186/s13045-021-01050-0 (PMC7927217; doi:10.1186/s13045-021-01050-0)
Supplement: Supplementary file 2 — Additional file 2: Table S1. The estimated odds ratio of high-frequency gene mutations as predictive marker in ICT pooled cohort [file 13045_2021_1050_MOESM2_ESM.pdf]

Supplementary Table 1. The estimated odds ratio of high-frequency gene mutations as predictive marker in ICT pooled cohort

Gene	Mutation Frequency	Odds Ratio (95% CI)			P-value
		Fixed effects (Mantel-Haenszel)	NSCLC	Melanoma	
KMT2 family	0.31	2.60 (1.66-4.07)	3.82 (1.65-8.85)	2.21 (1.29-3.78)	1.67E-04
TTN	0.64	2.08 (1.29,3.36)	2.11 (1.04-4.28)	2.06 (1.08-3.94)	2.85E-02
MUC16	0.58	1.86 (1.17,2.97)	2.13 (1.02-4.45)	1.71 (0.94-3.12)	1.36E-01
LRP1B	0.33	2.13 (1.37,3.31)	2.65 (1.19-5.89)	1.93 (1.13-3.29)	3.53E-03
USH2A	0.33	2.04 (1.31,3.18)	4.61 (1.95-10.86)	1.47 (0.86-2.51)	6.82E-03
CSMD3	0.31	1.76 (1.13,2.74)	1.43 (0.67-3.05)	1.97 (1.14-3.38)	1.70E-02
RYR2	0.27	2.18 (1.38,3.45)	2.65 (1.27-5.53)	1.92 (1.07-3.45)	3.41E-04
ZFHX4	0.24	2.39 (1.5,3.83)	4.97 (2.11-11.70)	1.66 (0.93-2.98)	3.45E-04
SYNE1	0.21	2.65 (1.6,4.39)	4.57 (1.52-13.72)	2.23 (1.25-3.97)	1.03E-03
RYR3	0.21	1.45 (0.87,2.43)	2.69 (0.91-7.92)	1.20 (0.66-2.17)	3.64E-01
KMT2C	0.15	3.48 (1.98,6.12)	4.67 (1.40-15.57)	3.15 (1.66-5.99)	5.46E-05
FAM135B	0.15	1.33 (0.75,2.37)	1.74 (0.59-5.12)	1.19 (0.60-2.37)	4.60E-01

P-value was estimated by Fisher's exact test
