# Supplementary material for: Genomic alterations in KMT2 family predict outcome of immune checkpoint therapy in multiple cancers
Source: J Hematol Oncol. 2021 Mar 2;14:39. doi: 10.1186/s13045-021-01050-0 (PMC7927217; doi:10.1186/s13045-021-01050-0)

SuppFigure 1

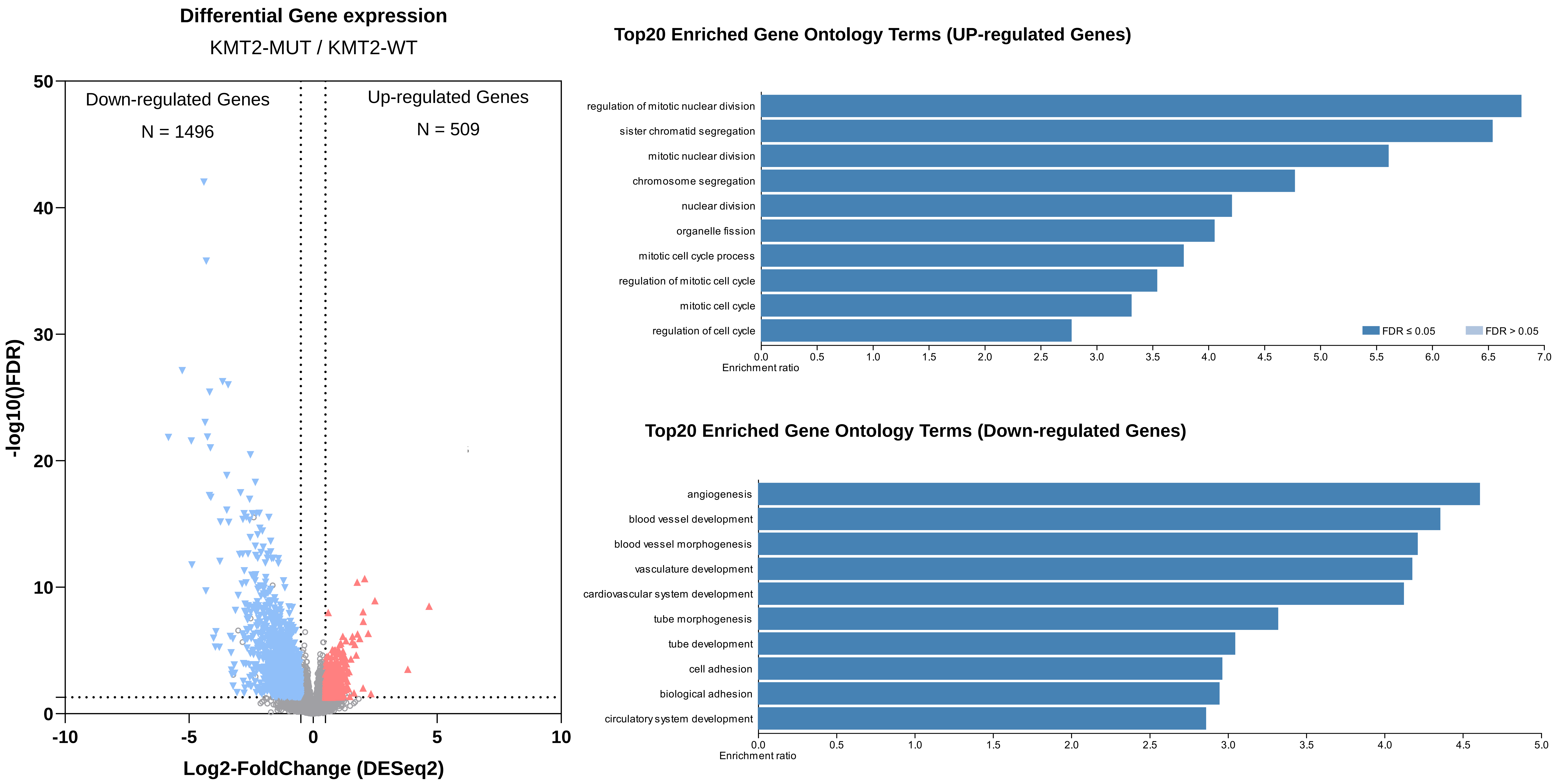

SuppFigure 2

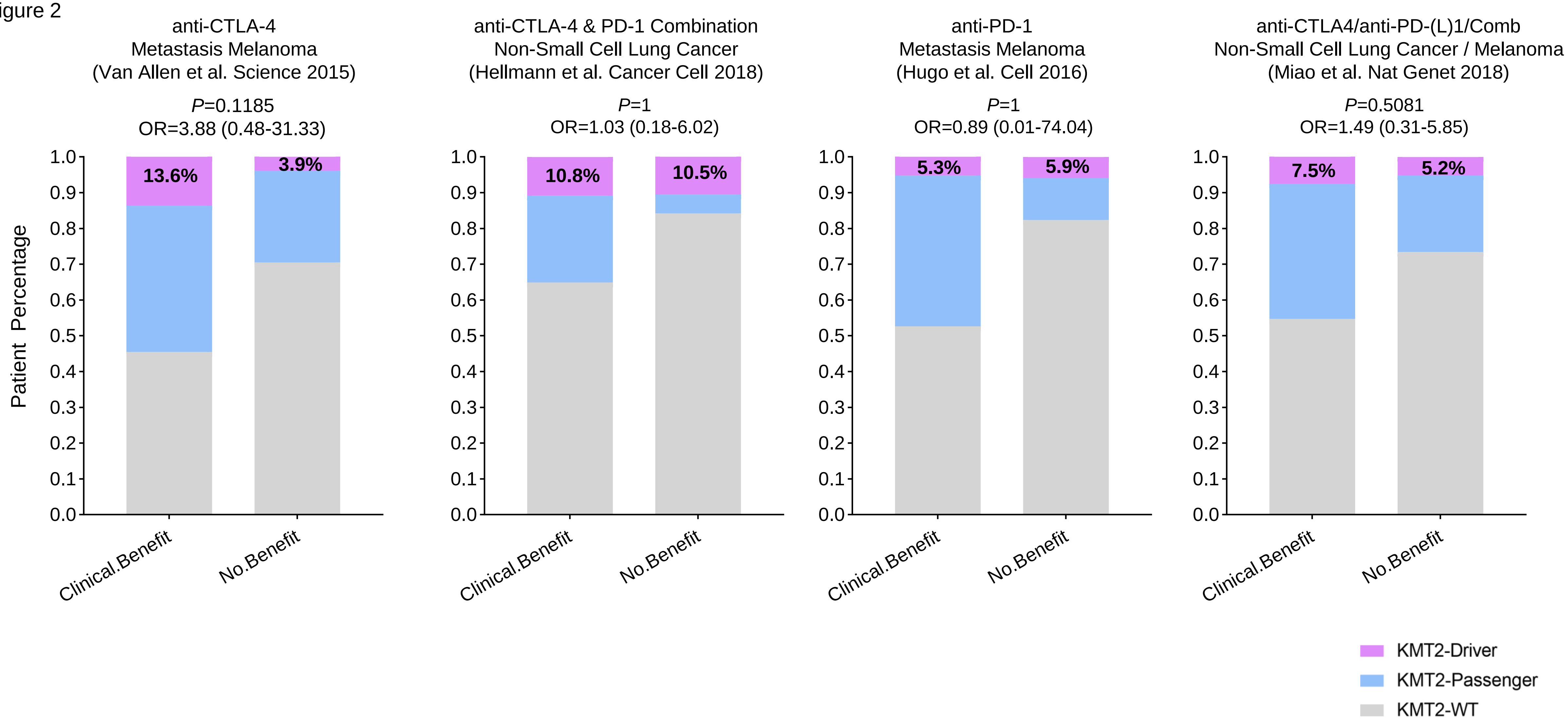

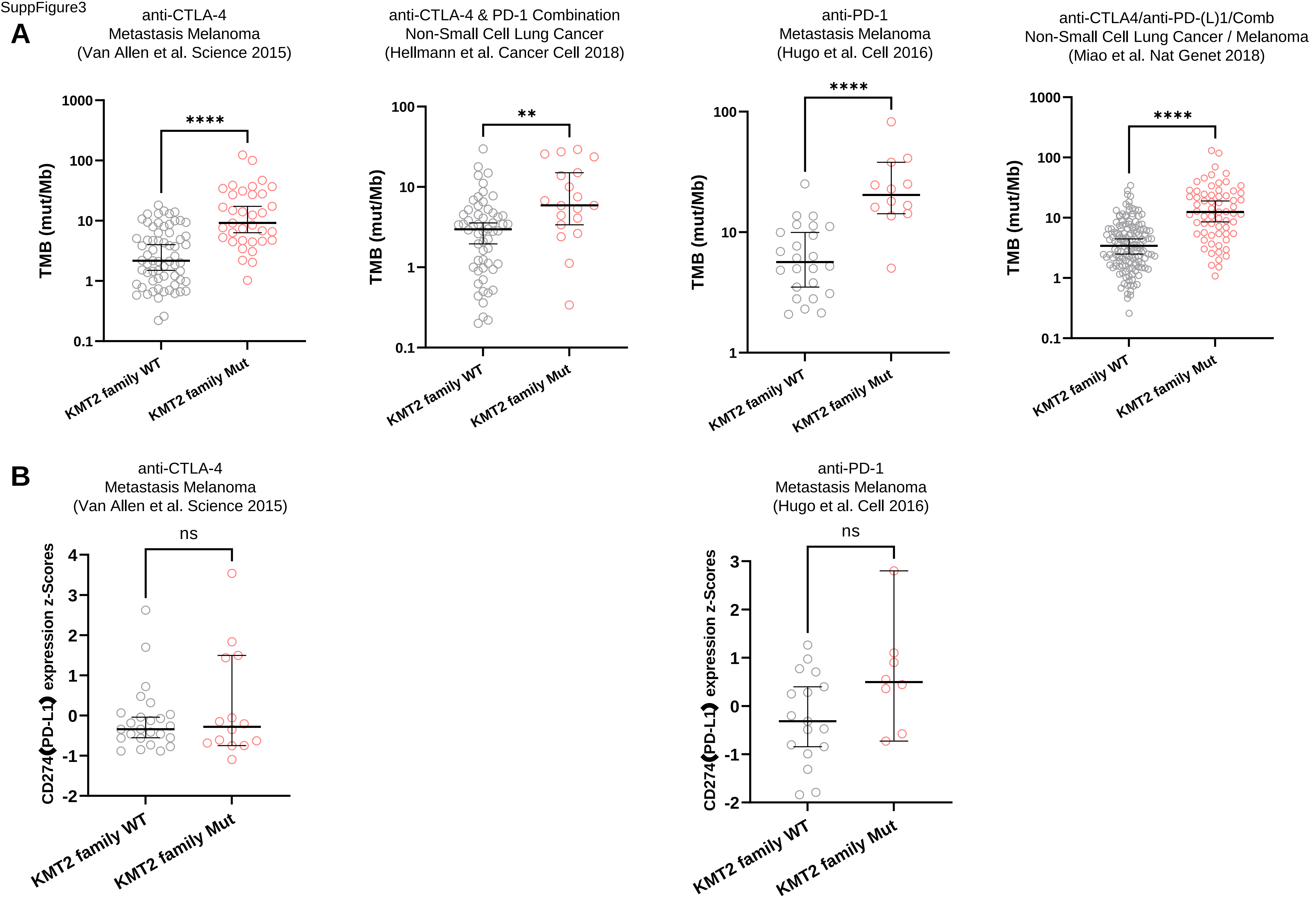
SuppFigure3
A
anti-CTLA-4
Metastasis Melanoma
(Van Allen et al. Science 2015)
anti-CTLA-4 & PD-1 Combination
Non-Small Cell Lung Cancer
(Hellmann et al. Cancer Cell 2018)
anti-PD-1
Metastasis Melanoma
(Hugo et al. Cell 2016)
anti-CTLA4/anti-PD-(L)1/Comb
Non-Small Cell Lung Cancer / Melanoma
(Miao et al. Nat Genet 2018)
TMB (mut/Mb)
****
**
KMT2 family WT
KMT2 family Mut
B
anti-CTLA-4
Metastasis Melanoma
(Van Allen et al. Science 2015)
anti-PD-1
Metastasis Melanoma
(Hugo et al. Cell 2016)
ns
CD274 (PD-L1) expression z-Scores
KMT2 family WT
KMT2 family Mut

SuppFigure4

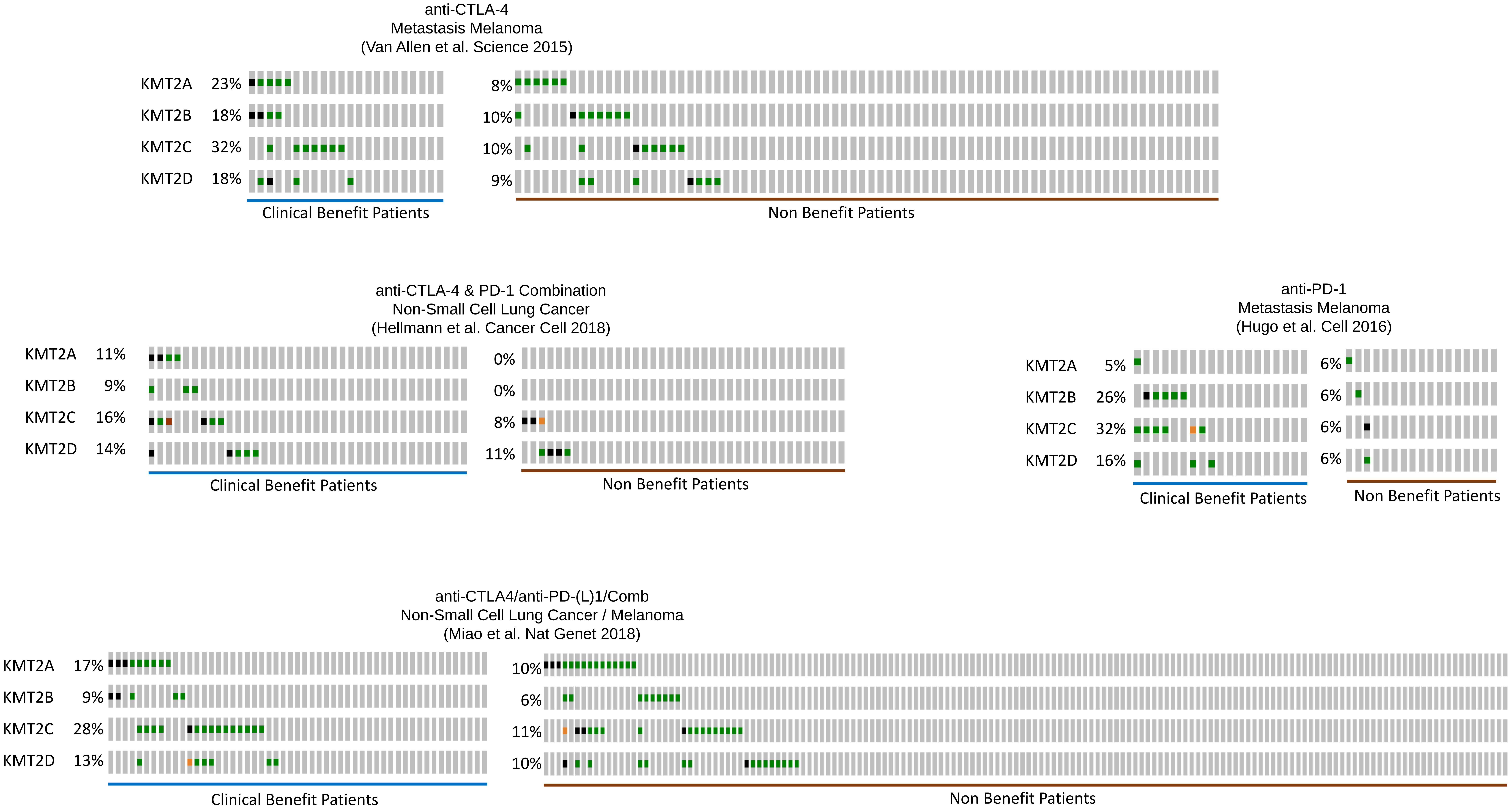

Supplement: Supplementary file 3 — Additional file 3: Figure S1. KMT2 mutations correlated with tumor microenvironment reorganization in metastasis melanoma. (A) Volcano plot of mRNA expression changes between metastasis samples harboring KMT2 mutation and wild-type. The x-axis specifies the fold-changes (FC), and the y-axis specifies the negative logarithm to the base 10 of the adjusted P values. Gray vertical and horizontal dashed lines reflect the filtering criteria. Red and blue dots represent genes expressed at significantly higher or lower gene levels, respectively. (B) Functional enrichment analysis based on gene ontology terms for differential expressed genes. Figure S2. No significant correlation was observed between predicted KMT2 driver mutations and ICT clinical outcomes. Histogram depicting proportions of harboring KMT2 family predicted driver mutations in clinical benefit and non-benefit groups of different ICT-treated patient cohorts. The P value and odds ratios were determined by Fisher's exact test. Figure S3. Association analysis between KMT2 family mutations and tumor immunogenicity in ICT cohorts. (A) The distribution plot of the tumor somatic mutation burden level between KMT2-MUT and KMT2-WT samples in all the ICT patient cohorts. (B) The distribution plot of the PD-L1 expression level between KMT2-MUT and KMT2-WT samples in all the ICT patient cohorts. The P value was determined by the Mann–Whitney test. Figure S4. The mutation pattern of KMT2 genes in ICT patient cohorts. Oncoprint schematics revealed the mutational pattern of the KMT2 family mutations in all the ICT patients cohorts involved in this study. Truncating mutations included nonsense, nonstop, splice site mutations, and frameshift insertion and deletion; Non-truncating mutations included missense mutations and inframe insertion and deletion. [file 13045_2021_1050_MOESM3_ESM.pdf]
